# Susceptibility of Males, but Not Females to Developing Femoral Head Osteonecrosis in Response to Alcohol Consumption

**DOI:** 10.1371/journal.pone.0165490

**Published:** 2016-10-27

**Authors:** Junya Shimizu, Shunichiro Okazaki, Satoshi Nagoya, Nobuyuki Takahashi, Kumiko Kanaya, Keisuke Mizuo, Hideki Hyodoh, Satoshi Watanabe, Toshihiko Yamashita

**Affiliations:** 1 Department of Orthopedic Surgery, Sapporo Medical University School of Medicine, Sapporo, Japan; 2 Department of Legal Medicine, Sapporo Medical University School of Medicine, Sapporo, Japan; 3 Department of Musculoskeletal Biomechanics and Surgical Development, Sapporo Medical University, Sapporo, Japan; Oregon Health and Science University, UNITED STATES

## Abstract

**Background:**

We previously reported that ethanol-containing liquid diet feeding induces osteonecrosis of the femoral head in male rats. Also, it was reported that a large amount of consumed ethanol and a long-term history of drinking were risk factors for osteonecrosis of the femoral head, and that the frequency of alcohol-induced osteonecrosis of the femoral head in males was much greater than in females. The higher incidence of alcohol-induced osteonecrosis of the femoral head could be due to either higher prevalence of alcohol drinking in males or due to their potential higher sensitivity to alcohol. The aim of the study is to investigate the influence of alcohol consumption and drinking period on the development of osteonecrosis of the femoral head in rats of both sex.

**Methods:**

All the experimental male rats were allocated to the male one-month ethanol drinking group (M1). Female rats were randomly divided into the female one- to five-months ethanol drinking groups (F1-5). All rats were fed a Lieber-DeCarli liquid diet containing 5% ethanol for one to five months.

**Results:**

One-month feeding with the ethanol-containing liquid diet resulted in the development of osteonecrosis of the femoral head in seven of twenty in the M1 group, but none in the F1 group, although the mean intake of ethanol per body weight in the M1 group was significantly lower than that in the F1 group. Furthermore, long drinking periods with a large amount of ethanol intake in the F2-5 groups did not induce osteonecrosis of the femoral head.

**Conclusion:**

The present study shows that lower alcohol consumption over short periods of time that were sufficient to induce osteonecrosis of the femoral head in males had no effect on females. Even with greater alcohol consumption and longer duration, females did not develop osteonecrosis of the femoral head. Therefore, unknown factors related to sex must be responsible for the development of this condition.

## Introduction

Recently, as more people have become addicted to alcohol, the incidence of alcohol-related diseases such as osteonecrosis of the femoral head (ONFH) has been increasing in patients [1]. In many cases, ONFH can result in the collapse of the femoral head and secondary degenerative osteoarthritis, with severe impairment of the patients [2, 3]. However, the pathogenesis of alcohol-induced ONFH has not been fully understood.

An addiction to alcohol and long drinking period were reported as a risk factor of alcohol-induced ONFH [4]. It has been also reported that the frequency of alcohol-induced ONFH in males was much greater than in females [5]. The higher incidence of alcohol-induced osteonecrosis of the femoral head could be due to either higher prevalence of alcohol drinking in males or due to their potential higher sensitivity to alcohol.

We previously reported an alcohol-induced ONFH animal model using male rats based on the feeding of an ethanol-containing liquid diet [6]. ONFH developed in male rats fed with 5% ethanol-containing liquid diet from only one-week feeding, and the incidence of ONFH in the 4-week ethanol-fed male rats was significantly higher than in the control group [6]. The aim of the present study is to investigate the influence of alcohol consumption and drinking period in the development of alcohol-induced ONFH in rats of both sex.

## Materials and Methods

All experiments observed the guidelines of the Ministry of Sports, Culture, Science, and Technology of Japan, and followed protocols approved by the Animal Ethics Committee of Sapporo Medical University (#14–039).

### Animals and liquid diet feedings

Wistar ST rats (n = 128) were obtained from the Sankyo Labo Service Co., Ltd. (Sapporo, Japan). All rats were housed individually in temperature-controlled conventional rooms. Light was kept on, between 07:00 am and 07:00pm. Forty male rats (6 weeks of age, 210–220 g) were randomly divided into the male control group (MC) and the male one-month ethanol drinking group (M1). Eighty-eight female rats (6 weeks of age, 145–160 g) were randomly divided into the female control group (FC) and the female one- to five-months ethanol drinking groups (FC: n = 20, F1: n = 20, F2: n = 12, F3: n = 12, F4: n = 12, F5: n = 12). The M1 and the F1-5 groups received the Lieber-DeCarli liquid diet (ORIENTAL YEAST Co.,Ltd., Tokyo, Japan) containing 5.0% (weight/volume) ethanol (35% ethanol-derived calories) for one to five months, as described previously [6, 7]. The MC and FC group rats were pair-fed the same liquid diet without ethanol (the ethanol was replaced with dextran-maltose isocalorically) as the control liquid diet for the same period as the M1 and F1 groups. All rats were fed the control diet ad libitum for 1 week prior to the start of experiments. To accustom the M1 and the F1-5 group rats to the ethanol-containing liquid diet, they were fed with an ethanol-containing liquid diet (1.0–4.0% ethanol) ad libitum for 1 week prior to the start of experiments. The body weights and liquid diet consumption of all rats were measured each day. The rats in the M1 and F1-5 groups were allowed unrestricted access to the ethanol-containing liquid diet until just before the euthanasia. The ingestion of the pair-fed MC and FC group rats was strictly limited to the amount ingested on the previous day by the pair-matched ethanol-fed M1 and F1 group rats, to ensure that the calorie intakes between the M1 and MC, and F1 and FC groups were the same.

All of the rats were euthanized one to five months after starting the 5% ethanol-containing diet feeding under three-type mixed anesthesia with 0.3 mg/kg of medetomidine, 4.0 mg/kg of midazolam, and 5.0 mg/kg of butorphanol from 9:00 am. The blood was collected from the portal vein at the time of euthanasia and centrifuged immediately. The supernatant was stored as platelet rich plasma (PRP) at -84°C until analysis. Both femoral heads and the liver were collected and immediately fixed with a 10% formalin-0.1 M phosphate buffer (pH 7.4).

### Blood alcohol concentration

Blood alcohol concentration was measured by GC-2014 gas chromatograph (SHIMADZU CORPORATION, Kyoto, JAPAN) with TurboMatrix 110 headspace injector (PerkinElmer Japan Co., Ltd., Yokohama, JAPAN). Headspace conditions: thermostatting 20 min at 60°C, needle transfer temperature 120°C. Ultra-pure grade helium was used as the carrier gas at a flow rate of 5.00 ml/min. The chromatographic column was DB-ALC2 (30 m, and 0.32 mm i.d, with a film thickness of 1.20 um, Agilent Technologies, Santa Clara, CA, USA). Injection temperature was 150°C; column conditions, 7 min at 50°C, FID 200°C.

### Biochemical assay

The plasma concentrations of aspartate aminotransferase (AST), alanine transaminase (ALT), triglyceride (TG), total cholesterol (TC) and high-density lipoprotein (HDL) were measured using a SPOTCHEM^®^ D system (ARKRAY, Inc., Kyoto, Japan) in accordance with the manufacturer’s instructions.

### Histopathology

A small section of the liver or femur was dissected and fixed with a 10% formalin in 0.1M phosphate buffer, pH 7.4. Bone samples of the femoral heads were decalcified with Kalkitox^™^ solution (Wako Pure Chemical Industries, Ltd. Osaka, Japan) and then neutralized with sodium sulfate buffer. The tissues were then sectioned into 2-μm-thick layers and processed for routine hematoxylin and eosin staining. Osteonecrosis was defined as the presence of diffuse empty lacunae in the bone trabeculae, accompanied by surrounding bone marrow cell necrosis, as described previously [8, 9, 10]. The percentage of empty lacunae in the bone trabeculae was evaluated microscopically. During an examination of the trabeculae at 200x magnification, 10 fields were randomly chosen and 50 bone lacunae were counted in each field, and then the percentage of the empty lacunae was determined [11, 12]. Liver vesicular steatosis was scored using NASH Clinical Research Network Scoring System based on the percentage of the total area affected, into the following categories: 0 (<5%), 1 (5–33%), 2 (34–66%) and 3 (>66%) [13].

### Statistical analysis

Data are expressed as means±SEM and were analyzed using GRAPHPAD PRISM 5.0f software for MAC OS X (GraphPad Software, Inc., La Jolla, CA, USA). Data were compared between the eight groups (MC, M1, FC and F1-5) using two-way ANOVA with Tukey’s multiple comparisons test; the percentage of empty lacunae between ONFH- and ONFH+ in the M1 group using the Mann-Whitney test; the incidence of ONFH between two groups using Fisher’s exact test. A *P* value of <0.05 was considered to be significant.

## Results

### Influence of ethanol consumption in the same periods

The MC, M1, FC and F1 groups were euthanized one month after starting administration of 5% ethanol-containing liquid diet to elucidate the influence of ethanol consumption and evaluate the incidence of ONFH. The body weights of the rats increased during the drinking period, so there is no influence of starvation. In the M1 and F1 groups, the mean intakes of ethanol per body weight in the groups were 12.17 and 14.74 g/kg/day, respectively (Table 1). The mean intake of ethanol per body weight in the F1 group was significantly higher than that in the M1 group (*P*<0.0001). The mean total amount of ethanol intake per body weight in the groups were 349.8 and 508.6 g/kg, respectively (Table 1). The mean total amount of ethanol intake per body weight in the F1 group was significantly higher than that in the M1 group (*P*<0.0001). Furthermore, the blood ethanol concentrations were 1.91 mg/mL in the M1 group and 2.25 mg/mL in the F1 group (Table 1).

**Table 1:** Characteristics of each group.

	MC	M1	FC	F1	F2	F3	F4	F5
**Mean intakes of ethanol per body weight (g/kg/day)**	**0.0±0.0** †	**12.17±0.23** ‡	**0.0±0.0** †	**14.74±0.24**	**14.12±0.29**	**13.29±0.27**	**12.76±0.21**	**12.74±0.20**
**Total amount of ethanol intake per body weight (g/kg)**	**0.0±0.0**	**349.8±3.36** §	**0.0±0.0**	**508.6±5.10** §	**804.5±7.94** §	**1130.0±15.27** §	**1442.0±14.97** §	**1726.0±10.91** §
**Blood-ethanol concentrations (mg/mL)**	**0.0±0.0**	**1.91±0.42** ¶	**0.0±0.0**	**2.25±0.15** ††	**1.25±0.21** ‡‡**,** §§	**1.20±0.23** ‡‡**,** §§	**1.18±0.21** ‡‡	**1.09±0.12** ‡‡**,** §§
**The incidence of ONFH**	**0/20**	**7/20** ¶¶	**0/20**	**0/20**	**0/12**	**0/12**	**0/12**	**0/12**

^†^ vs F1-5, *p* <0.001,

^‡^ vs MC, FC, *p* <0.01,

^§^ vs all of the other groups, p<0.0001,

^¶^ vs MC, FC, *p* <0.0001, vs F5, *p* <0.05,

^††^ vs MC, FC, F5, *p* <0.0001, vs F2-4, *p* <0.01,

^‡‡^ vs MC, *p* <0.001,

^§§^ vs FC, *p* <0.01,

^¶¶^ vs MC, FC, F1, *p* <0.01, vs F2-5, *p* <0.05.

**Fig 1:**
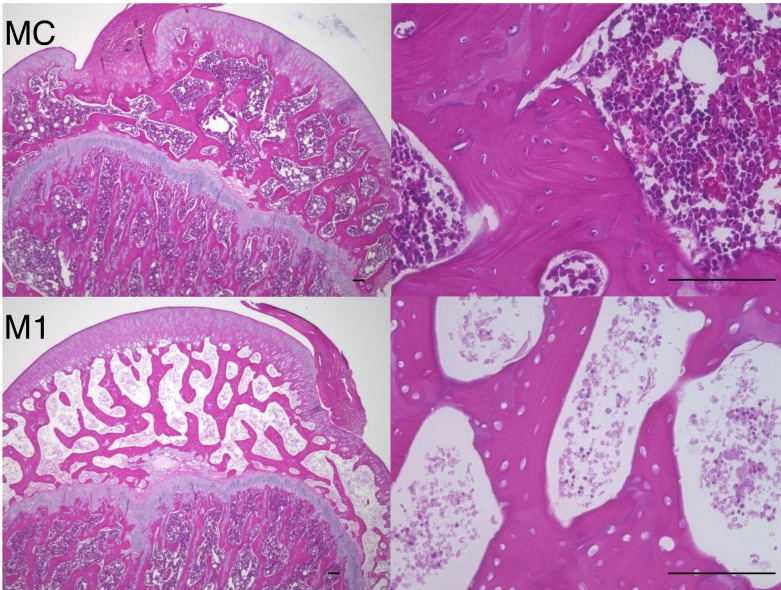
Histological appearance of hematoxylin and eosin stained femoral head. Typical images from the MC and M1 groups are shown. The diffuse presence of empty lacunae in the bone trabeculae accompanied by bone marrow cell necrosis was observed in the femoral head of M1 group. Scale bar: 100μm.

**Fig 2:**
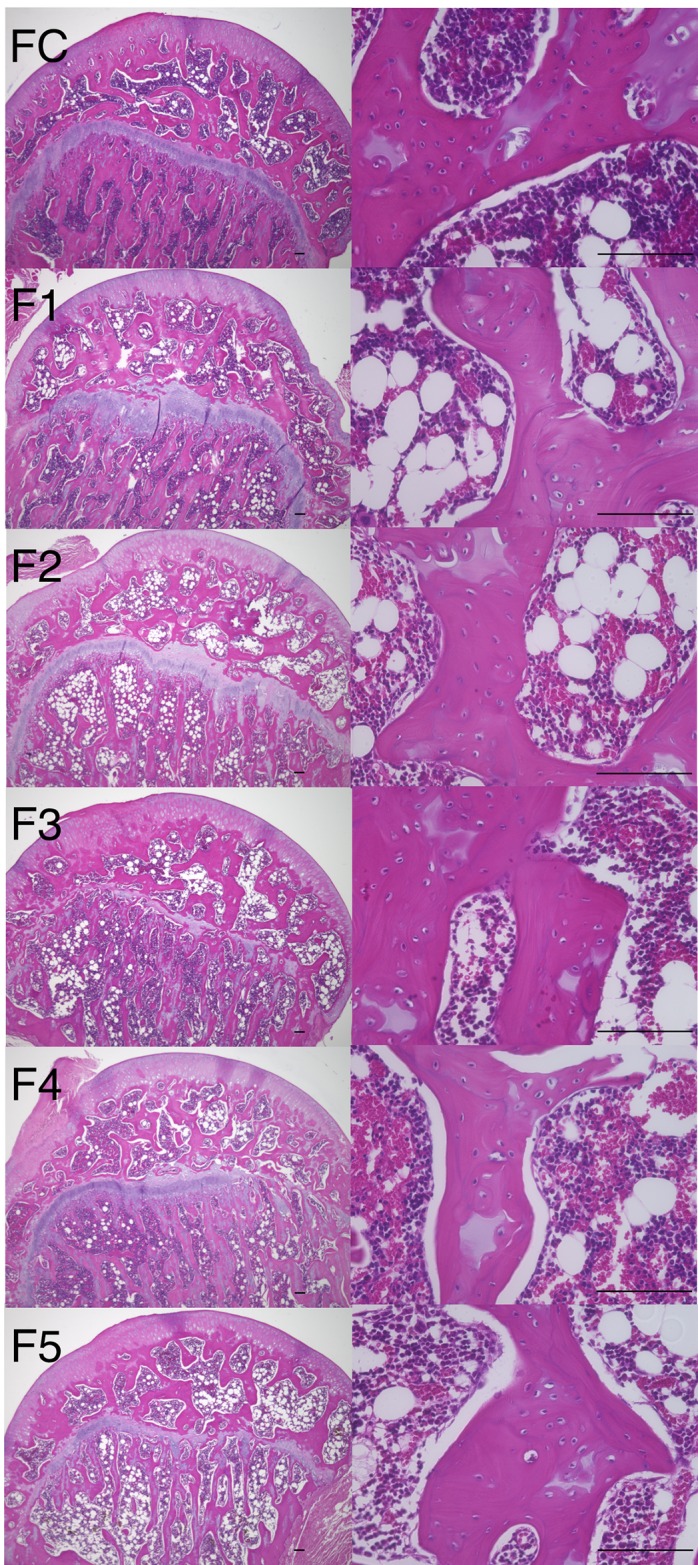
Histological appearance of hematoxylin and eosin stained femoral head. Typical images from the FC, F1, F2, F3, F4 and F5 groups are shown. Normal trabeculae and normal hematopoietic and fat cells were observed in the femoral head. Scale bar: 100μm.

**Fig 3:**
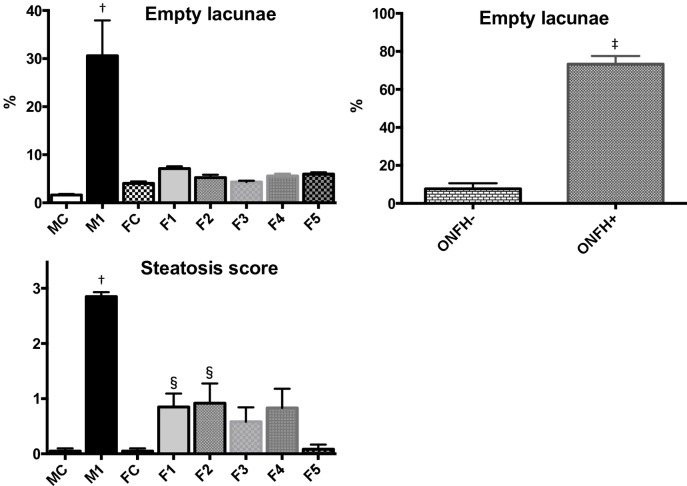
The percentage of empty lacunae in the femoral head and NASH steatosis score in the liver. The percentage of empty lacunae in the M1 group was significantly higher than in the other groups (p<0.0001). The percentage of empty lacunae in the M1 group with ONFH was significantly higher than in the M1 group without ONFH (ONFH- vs ONFH+: 7.62±0.82 vs 73.29±4.34, Fig 3). Hepatic steatosis score in the M1 group was significantly higher than in the other groups (p<0.0001). ^†^ vs MC, FC, F1-5, *p* <0.0001, ^‡^ vs ONFH-, *p* <0.0001, Mann-Whitney test, ^§^ vs MC, FC, *p* <0.05.

ONFH was observed in seven of twenty in the M1 group (Table 1). On the other hand, no ONFH was observed in the MC, FC and F1 groups (Table 1). The incidence of ONFH in the M1 group was significantly higher than in the MC, FC or F1 group (*P*< 0.01, Fisher’s exact test). In the necrotic area of the femoral heads in the M1 group, there are characteristic changes in histological appearance, like the presence of an accumulation of bone marrow cell debris in the medullary space, empty lacunae within the necrotic bone trabeculae, and bone marrow cells including adipocyte necrosis surrounding bone marrow cell necrosis (Fig 1). On the other hand, in the MC, FC and F1 groups, there were normal trabeculae and normal hematopoietic and fat cells in the femoral head (Figs 1 and 2). The percentage of empty lacunae in the M1 group was significantly higher than in the MC, FC and F1 groups (p<0.0001, Fig 3). The percentage of empty lacunae in the M1 group with ONFH was significantly higher than in the M1 group without ONFH (ONFH- vs ONFH+: 7.62±0.82 vs 73.29±4.34, *p* <0.0001, Mann-Whitney test, Fig 3).

**Fig 4:**
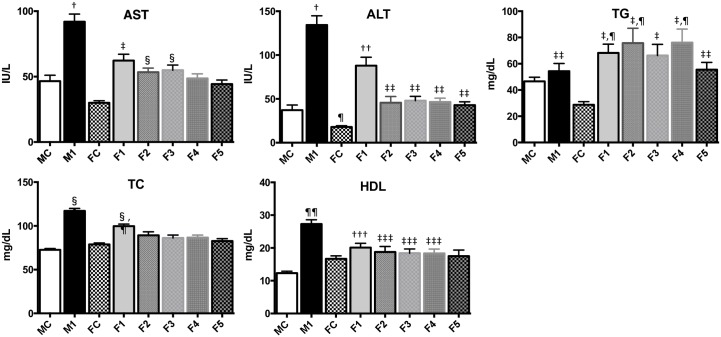
Plasma concentration of AST, ALT, TG, TC and HDL. In the M1 group, all the parameters except TG were significantly higher compared to the MC group (*P*<0.0001); AST, ALT and HDL were significantly higher compared to the F1 group (*P*<0.0001). ^†^ vs MC, FC, F1-5, *p* <0.0001, ^‡^ vs FC, *p* <0.0001, ^§^ vs FC, *p* <0.05, ^¶^ vs MC, *p* <0.01, ^††^ vs MC, FC or F2-5, *p* <0.0001, ^‡‡^ vs FC, *p* <0.01, ^§§^ vs MC, FC, F5, *p* <0.0001, vs F2-4, *p* <0.01, ^¶¶^ vs MC, FC, F1, 3–5, *p* <0.0001, vs F2, *p* <0.001 ^†††^ vs MC, *p* <0.0001, ^‡‡‡^ vs MC, *p* <0.05,

**Fig 5:**
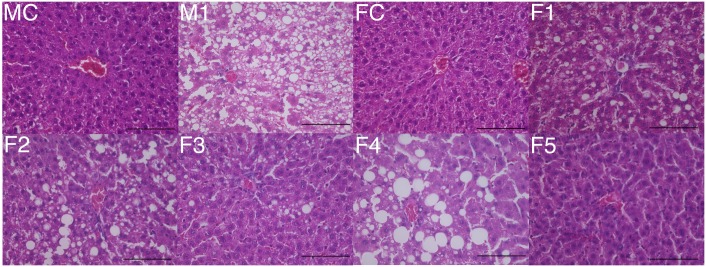
Histological appearance of hematoxylin and eosin stained liver. The M1 group liver shows moderate hepatocellular steatosis. Scale bar: 100μm.

Fig 4 shows the plasma concentration of AST, ALT, TG, TC and HDL. In the male groups, all the parameters except TG were significantly higher in the M1 group compared to the MC group (*P*<0.0001). In the female groups, all the parameters except HDL were significantly higher in the F1 group compared to the FC group (*p*<0.0001). In the M1 group, AST, ALT and HDL were significantly higher compared to the F1 group (*P*<0.0001). Fig 5 shows the histopathology of liver. In the MC and FC rats, the livers had the expected regular chord-like architecture. The liver in the M1 group showed moderate hepatocellular steatosis, and in the F1 group showed mild hepatocellular microsteatosis. The mean score of steatosis in the MC, M1, FC and F1 groups were 0.05, 2.85, 0.05 and 0.85, respectively (Fig 3). The degree of the hepatic steatosis in the M1 group appears to be significantly more severe than in the MC or F1 group (p<0.0001).

### Influence of long drinking periods in female rats

As described above, ONFH developed only in 4-week ethanol-fed male rats, but not in female rats. To elucidate the influence of drinking periods, F2-5 groups were euthanized two to five months after starting administration of 5% ethanol-containing liquid diet. In the F2-5 groups, the mean intakes of ethanol per body weight in the groups were 14.12, 13.29, 12.76 and 12.74 g/kg/day, respectively (Table 1). The mean total amount of ethanol intake per body weight in the F2-5 groups was 804.5, 1130.0, 1442.0, 1726.0 g/kg, respectively (Table 1). Those in the F2-5 groups were significantly higher than those in the FC, F1 or M1 group (*P*<0.0001). Furthermore, the blood-ethanol concentrations in the F2-5 groups were 1.25, 1.20, 1.18 and 1.09 mg/mL, respectively (Table 1). The blood-ethanol concentration in the F2-5 groups was significantly lower than that in the M1 or F1 group (*P*<0.0001).

No ONFH was observed in the F2-5 groups (Table 1). In the F2-5 groups, there were normal trabeculae and normal hematopoietic and fat cells in the femoral head as in the F1 group (Fig 2).

In the F2-5 groups, all the parameters except TG were significantly lower compared to the M1 group (*P*<0.0001, Fig 4). In the F2-5 groups, ALT showed significantly lower than in the F1 group. In the F3-5 groups, TC showed significantly lower than in the F1 group (P<0.05).

In the F2-5 groups mild hepatocellular microsteatosis (Fig 5) was observed. The mean scores of steatosis in the F2-5 groups were 0.91, 0.58, 0.83 and 0.08, respectively (Fig 3). The degree of the hepatic steatosis in the F2-5 groups appears to be significantly milder than in the M1 group (p<0.0001).

## Discussion

Hirota et al. reported that a large amount of consumed ethanol and a long-term history of drinking were risk factors for alcohol-induced ONFH [4]. Furthermore, Fukushima et al. conducted a nationwide epidemiologic survey in Japan and reported that the frequency of alcohol-induced ONFH in males was eight times greater than in females [5]. Therefore, it has been speculated that the sex difference in the incidence of alcohol-induced ONFH is caused by the fact that the male patients tend to drink more than the female ones. We previously reported that feeding a 5% ethanol containing liquid diet induced ONFH in male rats, and that ONFH was observed after one week of ethanol liquid diet feeding [6]. In the present study, however, the incidence of ONFH in the M1 group was significantly higher than that in the F1 group, although the mean intake of ethanol per body weight and the blood concentration of ethanol in the M1 group were both lower than that in the F1 group. Although the F2-5 group rats consumed a large amount of ethanol over long periods, ONFH did not develop, and liver dysfunction was also milder than in the M1 group. Consequently, these results suggest that a smaller amount of consumed ethanol and shorter term of drinking period in male than in female causes the development of alcohol-induced ONFH.

We previously reported that liver dysfunction might contribute to the development of corticosteroid/alcohol-induced ONFH [6, 14, 15]. In the present study, ethanol administration also led to liver dysfunction biochemically and histopathologically in the M1 and F1-5 groups. The M1 group developed more severe liver dysfunction than the F1-5 groups, although the mean intake of ethanol per body weight in the M1 group was lower than that in the F1-5 groups. Results of previous studies in rodents indicate that ethanol intake more easily induces liver damage in a female than in a male [16, 17]. However, on the contrary, it has been reported that females maintain a more robust inflammation response, and that chronic ethanol feeding more easily activated a DNA repair mechanisms that can activate an endogenous repair pathway in a female rat than in a male [18]. It has been also reported that the prevalence of cirrhosis in patients with chronic alcoholism was significantly higher among male patients than female [19]. Consequently, the present study suggests that a large amount of consumed ethanol and a long-term history of drinking alone were not risk factors for the development of alcohol-induced ONFH; other factors such as liver dysfunction are relevant to the development of alcohol-induced ONFH.

A limitation of this study was that we used rats of immature age compared to the clinical cases. Since mature/older rats do not adapt to an ethanol liquid diet, we used immature rats as described in previous reports [6, 7].

In conclusion, the present study shows that lower alcohol consumption over short periods of time that were sufficient to induce osteonecrosis of the femoral head in males had no effect on females. Even with greater alcohol consumption and longer duration, females did not develop osteonecrosis of the femoral head. Thus, further analyses are required to clarify the pathogenesis of alcohol-induced ONFH.

## Supporting Information

S1 Table Detailed experimental data. (XLSX)
